# Perivascular Epithelioid Cell Neoplasm of the Lung: A Patient Case Report

**DOI:** 10.1155/crip/9952483

**Published:** 2026-06-24

**Authors:** Calista Sha, Riona Park, Michael Esposito, Paul C. Lee

**Affiliations:** ^1^ Department of Thoracic and Cardiovascular Surgery, Long Island Jewish Medical Center, Northwell Health, Donald and Barbara Zucker School of Medicine at Hofstra/Northwell, Hempstead, New York, USA, hofstra.edu; ^2^ Department of Pathology, Zucker School of Medicine at Hofstra/Northwell, Manhasset, New York, USA, hofstra.edu

**Keywords:** case report, lung cancer, PEComa, perivascular epithelioid cell

## Abstract

Perivascular epithelioid cell tumors (PEComa) are a family of tumors characterized by epithelioid cells and are often found in perivascular spaces. Cases originating in the lungs are highly uncommon. We discuss the case of a pulmonary PEComa found in a 71‐year‐old male who had two instances of lung cancer 4 years prior. He had received a left lower lobectomy and a subsequent right upper lobectomy for separate primary Stage IA adenocarcinomas. His most recent surveillance imaging revealed a cystic lesion in the left upper lobe. A surgical course of treatment was recommended, and a wedge resection was performed. After immunohistochemical and genetic analysis, the lesion was characterized as a localized PEComa that was positive for Melan‐A and Cathepsin‐K on IHC, although negative for HMB‐45. The current case contributes to the expanding histopathologic profile of these lesions.

## 1. Introduction

Perivascular epithelioid cell tumors (PEComas) are rare mesenchymal tumors characterized by perivascular epithelioid cells (PECs) and are found in various parts of the body. This broad range of tumors includes angiomyolipomas (AML), lymphangioleiomyomas (LAM), clear cell tumors of the lung (CCTL), and other unspecified PEComas from visceral sites, bones, and soft tissues [1].

PEComas originating in the lung are uncommon and scarcely reported. These are typically found incidentally in adults and more commonly occur in women than in men [2]. They are predominantly found in the retroperitoneum and gynecologic tract, but have been observed to involve the uterus, prostate, urinary bladder, and breast tissue as well [3–6]. PEComas that arise from the lungs are the pulmonary LAM and CCTL. Diagnostic challenges arise from the primarily indolent and asymptomatic development course [7].

Here, we report a rare PEComa of pulmonary origins in a patient with a history of lung cancer demonstrating unique immunohistochemical properties.

## 2. Case presentation

A 71‐year‐old male with a history of smoking and lung cancer was first introduced to our center 4 years prior for surgical consultation of bilateral lung nodules. He had a medical history of hyperlipidemia and rheumatoid arthritis. For the lung nodules, an elective left lower lobectomy was first performed for a Stage IA2 adenocarcinoma. A right middle lobectomy was subsequently done for a separate primary Stage IA1 adenocarcinoma. The patient tolerated both procedures well and consistently followed up for surveillance.

During the 4th year of follow‐up, a chest CT demonstrated a 2.2 cm cystic lesion in the left upper lobe. To further evaluate this lesion, a PET/CT was performed and nodular foci up to 4 mm along the cyst posterior wall was visualized, without FDG‐avidity. Despite an asymptomatic presentation, the lesion raised concern for malignancy as either recurrent disease or a new cystic adenocarcinoma. Despite two prior lobectomies, the patient had adequate performance status of pulmonary reserve. A surgical route was offered, and a robot‐assisted thoracoscopic wedge resection of the left upper lobe was performed.

**Figure 1:**
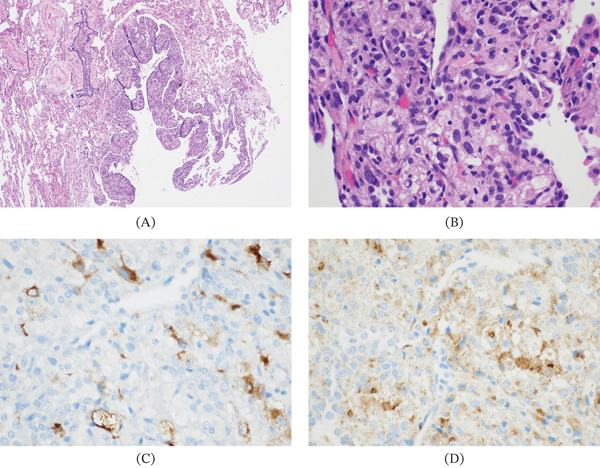
Histological staining of pulmonary PEComa. (A) H&E stain of cystic lesion from left lung (magnification 40×). (B) H&E staining granular cells (magnification 400×). (C) Positive expression of Melan‐A (magnification 400×). (D) Positive expression of Cathepsin‐K (magnification 400×).

Microscopy with hematoxylin and eosin (H&E) stains was used to examine the cystic lesion within the wedge sample (Figure 1A). There was a focal nodular and interstitial proliferation of uniform cells with granular cytoplasm (Figure 1B). There was mild focal atypia, but neither mitotic activity nor necrosis was seen. Immunohistochemistry (IHC) showed focal positivity for both Melan‐A (Figure 1C) and Cathepsin‐K (Figure 1D), whereas cells were negative for HMB‐45, SMA, desmin, TTF‐1, PAX‐8, CAM 5.2, CD34, ERG, synaptophysin, INSM‐1, CD117, AE1/3, CD1a, S100, CD163, and EMA.

The morphologic and immunohistochemical profile of the tumor correlated with a benign PEComa. A TFE3 fluorescence in situ hybridization (FISH) test was performed, with negative results, which confirmed the PEComa diagnosis. The lesion measuring 0.3 × 0.3 cm demonstrated localized activity, corresponding with benign PEComa behavior and favorable prognosis [8].

The patient is doing well with no evidence of recurrence and will continue regular follow‐ups.

## 3. Discussion

The origins of PEComas remain unclear, but there have been several hypotheses proposed. One hypothesis suggests that PECs are derived from the undifferentiated cells of the neural crest, enabling expression of both smooth muscle and melanocytic phenotypes. Others suggest that PECs have myoblastic and smooth muscle origins but have developed molecular alterations that cause expression of melanogenesis and melanocytic markers [9]. PEComa is molecularly characterized by mutations involving the tuberous sclerosis complex (TSC) and mTOR pathway, and more recently by TFE3 gene translocations [10].

PEComas generally share morphological and immunohistochemical features, which aid in diagnosing this broad group of neoplasms. Typical features include an epithelioid appearance with a clear to granular cytoplasm, and a centrally located nucleus. The most sensitive markers for the diagnosis of PEComa are HMB‐45 and Melan‐A, though expression of Cathepsin‐K or vimentin has been observed as well [1, 8, 11, 12].

Although Melan‐A expression was seen, HMB‐45 and all muscle markers were negative on IHC in our case, which is uncommon among conventional PEComas. A single melanocytic marker may be sufficient for diagnosis, since myogenic differentiation can be completely absent (Table 1) [14]. HMB‐45 and muscle marker coexpression has been used as the basis of diagnosis for PEComas, but some suggest Melan‐A may be a more sensitive marker [21]. Although PECs can express both melanocytic and smooth muscle markers, immunophenotypes vary and preferential expression of one can occur. In our case, Melan‐A and Cathepsin‐K were expressed without muscle markers. This correlates with the epithelioid appearance of tumor cells in our case, whereas PECs with dominant expression of muscle markers may appear more spindle‐shaped (Figure 1) [22].

**Table 1:** Case reports of PEComas with negativity for all tested muscle markers on IHC.

Case	Patient	All positive IHC markers	Negative muscle IHC markers	TFE3 FISH
1 (current case)	71/M	Melan‐A, Cathepsin‐K	SMA, Desmin	Negative
2 [13]	63/M	HMB‐45, Melan‐A, TFE3, Vimentin	SMA	N/A
3 [10]	56/M	TFE3	SMA, Desmin	Positive
4 [14]	39/M	HMB‐45, TFE3	SMA, MSA, Desmin, Caldesmon	Positive
5 [15]	28/F	HMB‐45, CD34, Vimentin	SMA, Desmin, MyoD1	N/A
6 [16]	26/F	TFE3, Melan‐A	SMA, Desmin	Negative
7 [16]	64/M	TFE3, HMB‐45, Melan‐A, CD10	SMA, Desmin	Negative
8 [17]	54/M	Melan‐A, TFE3, Vimentin	SMA, Caldesmon, myogenin, *α*‐SMA	Negative
9 [18]	59/M	Melan‐A, S100	SMA, Desmin	N/A
10 [19]	64/F	HMB‐45, Cathepsin‐K, TFE3	Desmin	Positive
11 [20]	14/F	HMB‐45, TFE3, Vimentin	SMA, Desmin, MSA	Positive

Abbreviations: IHC, immunohistochemistry; N/A, not available; SMA, smooth muscle actin.

Although predominantly benign, anomalies in pulmonary PEComa behavior have been observed. Case reports have shown how pulmonary PEComas can be malignant or mimic malignant behavior in terms of tumor size, vascularity, and extent of invasion [15, 23, 24]. They have also been reported to recur with and without metastases up to 10 years following primary resection [25, 26]. The guidelines in determining malignancy are unspecific to PEComas of pulmonary origins, and assessment of all subtypes relies on multicase analyses of mostly extrapulmonary PEComas. The standard Folpe criteria were determined by studying PEC tumors involving soft tissues and the gynecologic tract, excluding behavior of tumors originating from the lungs [8]. In 2014, Schoolmester et al. [27] proposed categorical updates, but focused their review on gynecologic PEComas. More recently, the 2020 WHO classification of soft tissue tumors divides PEComas into benign and malignant types by utilizing the Folpe criteria where at least two qualifications (tumor size ≥ 5 cm, infiltrative pattern, high nuclear grade and cellularity, high mitotic rate [≥ 1/50 high‐power field], necrosis, and lymphovascular invasion) must be met for malignant classification of all nongynecologic PEComas [28].

Cathepsin‐K expression with simultaneous negativity for HMB‐45 and muscle markers is more often seen in a different class of neoplasms. Referred to as “TFE3‐rearranged PEComa‐like neoplasms,” these tumors contain gene translocations or fusions involving TFE3, a member of the MiT family (MiTF) of transcription factors [29]. The immunophenotype covered in our case initially seemed to complement this group, but FISH analysis did not detect any TFE3 abnormalities or rearrangements, supporting conventional PEComa diagnosis.

TFE3‐rearranged PEComas are believed to use alternative means of pathogenesis. Although typical PEComas with mTOR dysregulation arise from TSC1/2 mutations, TFE3‐rearranged PEComas are more closely related to the MiTF spectrum of tumors and contain TFE3 gene translocations, which can mediate Cathepsin‐K expression [10, 30]. Argani et al. [16, 29] analyzed 25 cases of TFE3‐rearranged PEComas, of which, most tested cases were positive for Cathepsin‐K, four cases lacked HMB‐45 expression, and muscle markers were predominantly negative. Minimal immunoreactivity for muscle markers has been considered a distinguishing trait of TFE3‐rearranged PEComa‐like neoplasms [16, 29]. It has even been estimated that 20% of diagnosed PEComas that are completely negative for muscle markers may fall into this group [8, 16]. Challenging this characterization are others who have identified TFE3‐rearranged PEComas that contrarily expressed muscle marker (SMA) and lacked HMB‐45, prompting the need for further investigation to better distinguish the two [16, 31].

Typical PEComas and those harboring TFE3 rearrangements deserve further study to improve clinical discernment and avoid misdiagnosis. They respond to different therapeutic targets, and TFE3‐rearranged neoplasms may correspond with more aggressive behavior in terms of higher rates of systemic metastasis, recurrence, and death [29]. As with our case, when encountering anomalies, we suggest the diagnostic workup should comprehensively include molecular or genetic confirmation in cases lacking muscle marker expression, which is not always pursued (Table 1). There have been cases of pulmonary PEComa tumors that aberrantly lack HMB‐45 expression, with even fewer evaluating TFE3 IHC or molecular testing [10, 26–29]. Additionally, PEComas with TFE3 expression have been shown to lack the genetic rearrangement, indicating that TFE3 IHC is insufficient to make the distinction if interpreted without genetic confirmation [16, 17, 31].

PEComas may be detected through imaging and are ultimately confirmed through biopsy or surgical resection and immunohistochemical analysis. With a previous history of lung cancer, our patient was consistently monitored with annual chest CT scans. Following several years of observation, the new lung nodule raised suspicions for a new primary lung cancer or recurrence and was treated accordingly through surgery. However, upon confirming PEComa diagnosis, we believe that its development was independent of the patient′s prior lung cancers. Without annual chest CT scans, the PEComa may have advanced further despite its benign and asymptomatic development at the time. Our patient′s case reinforces the challenge of diagnosing PEComa, especially in patients who are not being followed closely, due to its indolent course of development. Additionally, PEComa immunophenotypes appear inconsistent, genetic testing is performed variably, and malignancy assessment is derived from studies of other PEComa subtypes. Our case report emphasizes the necessity for further research to better diagnose and manage patients with PEComas in the lung.

## 4. Conclusions

PEComas are usually benign epithelioid cell tumors that arise in various perivascular spaces, with limited documentation covering those of pulmonary origin. With a history of lung cancer and the development of PEComa in the lung, our patient′s case is extremely rare. Our report also contributes to the expanding immunohistochemical profile associated with PEComas (HMB‐45 negativity without TFE3 rearrangement). Without surveillance imaging, the patient′s PEComa may have been left undiagnosed, risking further progression and advancement. This case underscores the importance of recognizing PEComas in the assessment of incidental lung nodules and the need for a better understanding of the characterization, diagnosis, and treatment of this tumor.

## Funding

No funding was received for this manuscript.

## Ethics Statement

Informed consent from the participating individual was obtained. Ethical review and approval were waived for this study due to the Northwell Health Feinstein Institute′s Human Research Protection Program guidance document for case reports.

## Conflicts of Interest

The authors declare no conflicts of interest.

## Data Availability

Research data are not shared.
